# Receiver-Side TCP Countermeasure in Cellular Networks

**DOI:** 10.3390/s19122791

**Published:** 2019-06-21

**Authors:** Pingping Dong, Kai Gao, Jingyun Xie, Wensheng Tang, Naixue Xiong, Athanasios V. Vasilakos

**Affiliations:** 1 College of Information Science and Engineering, Hunan Normal University, Changsha 410081, China; ppdong@csu.edu.cn (P.D.); jingyunxie1995@gmail.com (J.X.); Tangws@hunnu.edu.cn (W.T.); 2 Hunnan Provincial Key Laboratory of Intelligent Computing and Language Information Processing, Hunan Normal University, Changsha 410081, China; 3 College of Automotive and Mechanical Engineering, Changsha University of Science & Technology, Changsha 410114, China; 4 Hunan Key Laboratory of Smart Roadway and Cooperative Vehicle-Infrastructure Systems, Changsha 410114, China; 5 College of Intelligence and Computing, Tianjin University, Tianjin 300350, China; xiongnaixue@gmail.com; 6 Department of Computer Science, Electrical and Space Engineering, Lulea University of Technology, 93187 Skelleftea, Sweden; th.vasilakos@gmail.com

**Keywords:** cellular networks, receiver-side, congestion control

## Abstract

Cellular-based networks keep large buffers at base stations to smooth out the bursty data traffic, which has a negative impact on the user’s Quality of Experience (QoE). With the boom of smart vehicles and phones, this has drawn growing attention. For this paper, we first conducted experiments to reveal the large delays, thus long flow completion time (FCT), caused by the large buffer in the cellular networks. Then, a receiver-side transmission control protocol (TCP) countermeasure named Delay-based Flow Control algorithm with Service Differentiation (DFCSD) was proposed to target interactive applications requiring high throughput and low delay in cellular networks by limiting the standing queue size and decreasing the amount of packets that are dropped in the eNodeB in Long Term Evolution (LTE). DFCSD stems from delay-based congestion control algorithms but works at the receiver side to avoid the performance degradation of the delay-based algorithms when competing with loss-based mechanisms. In addition, it is derived based on the TCP fluid model to maximize the network utility. Furthermore, DFCSD also takes service differentiation into consideration based on the size of competing flows to shorten their completion time, thus improving user QoE. Simulation results confirmed that DFCSD is compatible with existing TCP algorithms, significantly reduces the latency of TCP flows, and increases network throughput.

## 1. Introduction

With the boom of connected vehicles and other mobile devices [1,2,3], users generate ever-increasing demands on cellular networks, like 5G and LTE. However, long delays exist when accessing the Internet through wireless mobile networks [4,5,6,7]. One of the main reasons is unnecessarily large-sized buffers at intermediate routers and end hosts due to the low price of memory.

Owing to the excessive buffer space, the widely deployed transmission control protocol (TCP) implementations, which are loss-based congestion control algorithms, such as NewReno [8] and CUBIC  [9], will rarely suffer a loss even if they fully utilize the bandwidth. Thus, the TCP sender will keep increasing the amount of in-flight data. This results in up to several seconds of round trip delay [10,11,12,13]. However, the phenomenon does not cause critical problems when only one flow utilizes the buffer, as short flows will not build up queue and throughput matters with long flows.

However, smart phones are becoming more and more powerful and are usually equipped with several core processors. Thus, users expect them to perform multitasking simultaneously. If both long flows and short flows are coexisting, the short flows can experience huge flow completion time (FCT) when the buffers are filled with the packets belonging to the long flows [14].

To tackle this problem, researchers have proposed some algorithms. The sender-oriented approaches [7] proposed to utilize the round trip time (RTT), e.g., TCP Vegas [15], or the bandwidth-delay product (BDP), rather than the packet loss event, to control the congestion window (cwnd) in this buffer bloat cellular networks. However, the delay-based approaches may suffer from bandwidth starvation when they coexist with loss-based approaches. In addition, some researchers focus on the AQM (AQM) at intermediate routers to throttle the number of packets that stay in the buffer, such as Codel [16], PIE [17] and fq_Codel [18]. However, few intermediate routers enable AQM in practice [6], and it still remains unclear how quickly these AQM schemes will be deployed in practice including cellular networks, e.g., 5G and LTE networks.

Other works focus on the receiver side and the receiver-oriented approaches work with flow control which adjust their advertised receive window (rwnd) to limit the amount of in-flight data, like Dynamic Receiver Window Adjustment (DRWA)  [4,5] and Receiver-side TCP Adaptive queue Control (RTAC)  [6]. In cellular networks, base stations typically have a separate buffer space for each user. Thus, one of the main advantages of the receiver-oriented approach is that the receiver based mechanism will not influence the performance of other users. In addition, it can be implemented without the intervention of service providers and can quickly and easily be deployed by updating the firmware of the user’s device.

Although existing receiver-oriented mechanisms can alleviate the problem, the short flows can also show poor performance with the existing algorithms when they are competing with long-lived flows, i.e., a user playing an online game and, at the same time, downloading a song in the background, because there is no service differentiation between TCP flows. However, most of the TCP sessions in today’s Internet is constituted by short flows (e.g., web requests) [19]. Thus, it becomes of critical importance to enhance the performance of TCP in mobile networks to improve the quality of experience.

To this end, we proposed a novel receiver-oriented approach, named a Delay-based Flow Control algorithm with Service Differentiation (DFCSD), to mitigate the problem described above and improve the performance of both short flows and long flows in cellular networks. In DFCSD, the receiver controls the rwnd in a TCP-friendly manner and is automatically suitable for a certain application based on the TCP fluid model to achieve both performance improvement and latency reduction.

Note that DFCSD limits the sending rate through rwnd, which is calculated irrelevant to the congestion control algorithms at the sender side. Furthermore, it works effectively only when rwnd is smaller than cwnd, thus avoiding throughput degradation. We show that DFCSD successfully prevents long delays and achieves good performance under resource competing environments. The main contributions of this paper are as follows:

- To improve the FCT of short flows, we developed a DFCSD algorithm, which can effectively alleviate the long delays caused by the oversized buffer, is compatible with existing TCP variants, and can fairly share resource with conventional receivers.
- A key challenge in the proposed DFCSD algorithm was the calculation of the advertised window for each competing flow to maximize the network utility, as well as guaranteed completion time of short flows. To this end, this paper advises different rwnd for different flows, utilizes the idea of TCP fluid model, and takes into account flow characteristics, i.e, the flow size.

## 2. Related Works

**Server-oriented end-to-end congestion control protocols:** Since a large part of mobile traffic is constituted by TCP flows, TCP congestion control is always one of the hottest research topics, and its performance is critically important [7,20]. So far, numeric TCP variants have been proposed, which fall into three categories, namely, loss-based, delay-based, and combined loss- and delay-based. TCP Taho [21], TCP Reno, and TCP NewReno [8] are among the early approaches and are loss-based congestion control algorithms. Highspeed TCP (HSTCP) [22] and CUBIC [9] modify the window growth mode to quickly achieve high network utilization. Among them, CUBIC is the default congestion control algorithm in the current Linux kernel.

Delay-based protocols (e.g., TCP Vegas and FAST TCP [23]) detect the network congestion and adjust the cwnd based on RTT. The delay-based variants can react to the network congestion more quickly compared to the loss-based mechanisms  [24] and are capable of limiting the standing queue size and decreasing the amount of packets that are dropped in the eNodeB. However, the delay-based approaches will suffer from significant throughput degradation when competing with loss-based algorithms, e.g., TCP-Reno [25].

In addition, Compound TCP (CTCP) [26] incorporates the delay-based component into the loss-based TCP congestion avoidance algorithm. TCP Bottleneck Bandwidth and RTT (TCP BBR)  [27] estimates both bottleneck bandwidth and RTT delay and uses a distributed control loop to try to verge on the optimum to fully utilize the network while maintaining a small queue. Recently, a number of new algorithms have also been proposed, like Low Extra Delay Background Transport (LEDBAT)  [28] and TCP Binary Increase Congestion control (BIC)  [29]. However, these mechanisms are mainly designed for the wired network and are not suitable for highly variable cellular networks.

Meanwhile, some TCP variants offering differentiation among flows have been proposed. TCP Nice [30] aims to reduce the interference inflicted by background flows on foreground flows by modifying TCP congestion control to be more sensitive to congestion than traditional protocols by detecting congestion earlier, reacting to it more aggressively, and allowing much smaller effective minimum cwnds. TCP TS-Prio [31] describes a simple method to differentiate services based on the congestion control parameter configuration, i.e., the sliding window configuration of a TCP server and a simple priority marker. This algorithm requires that the TCP server recognizes these priorities, and the queue management policy should be RED (Random Early Detection) or similar. The authors in Reference  [32] proposed an approach which automatically prioritizes short (interactive) transfers by basing the priority of packets on the TCP connection window to achieve the goal of reducing congestion-induced delays for interactive applications using service differentiation mechanisms. However, this needs the support of the intermediate router.

Fortunately, several congestion control algorithms have been proposed aiming to improve the TCP performance in cellular networks. C2TCP [7] classifies the network into “good-condition” and “bad-condition” based on the idea of Codel [16]. It increases the cwnd when the network is in the “good-condition” and sets cwnd to one when the network is in the “bad-condition”. Sprout [10] adjusts its cwnd by predicting the bandwidth of the mobile network while Verus [11] calculates the cwnd based on the current network delay.

**Active queue management (AQM) schemes:** These algorithms aim to throttle the number of packets those stay in the buffer at intermediate routers. The main idea behind these AQM schemes is dropping packets at the router of bottleneck links so that the sender can slow down its sending rate. Traditional AQM algorithms, like RED [33], BLUE [34], and AVQ [35], have many tuning parameters, making them hard to implement [10,36]. To solve this problem, Codel [16], PIE [17], and fq_Codel [18] have recently been proposed. However, these improved AQM schemes still have an important issue when applied to cellular networks. On one hand, these schemes have the same setting for all applications, which is not the case in the real network where every application may have different delay or throughput requirements. On the other hand, it still remains unclear how quickly these AQM schemes will be deployed in practice included in cellular networks, e.g., 5G and LTE networks.

**Receiver-oriented protocols:** The receiver-oriented approaches work with flow control, which control the sending rate by the advertised rwnd. Until now, serveral algorithms have been proposed. In DRWA [4,5], the receiver increases rwnd when the current RTT is close to the minimum RTT and decreases it when RTT becomes larger, aiming to keep RTT close to its minimum RTT. RTAC [6] integrates the AQM into the loss-based congestion algorithms and is implemented at the receiver side. However, neither of the schemes provides service differentiation. In cellular networks, base stations typically have a separate buffer space for each user. Thus, one of the main advantages of the receiver-oriented approach is that the receiver based mechanism will not influence the performance of other users. In addition, it can be implemented without the intervention of service providers and can quickly and easily be deployed by updating the firmware of the user’s device. Based on the above analysis, we proposed a receiver-oriented approach named DFCSD, which employs the TCP fluid model and service differentiation to maintain the network throughput while improving the FCT. We compare DFCSD with both DRWA and RTAC in Section 5.

## 3. Motivation

In this section, we demonstrate the conducted empirical studies based on ns-2 (version 2.35) patched with LTE-Module to analyze the root reason why current TCP algorithms fail to provide satisfactory performance and present the design objectives.

**Figure 1:**
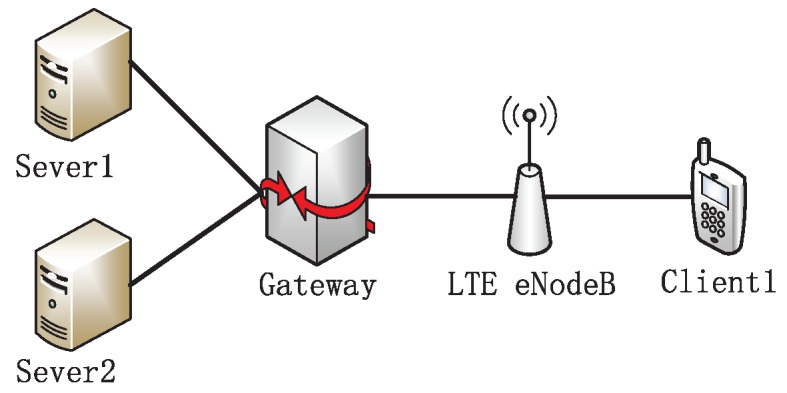
Simulation topology.

The topology is shown in Figure 1, where the UE (User Equipment) is connected to the eNodeB, and the eNodeB is attached to a gateway node through the Ethernet with 125 Mbps bandwidth and a 2 ms delay, ensuring it is not the bottleneck of the testbed. The UE downloads files from the server located in the wired side through FTP. The default buffer size of the base station is 50 packets. The size of the long flow and short flow is 10 MB and 128 KB, respectively.

**Figure 2:**
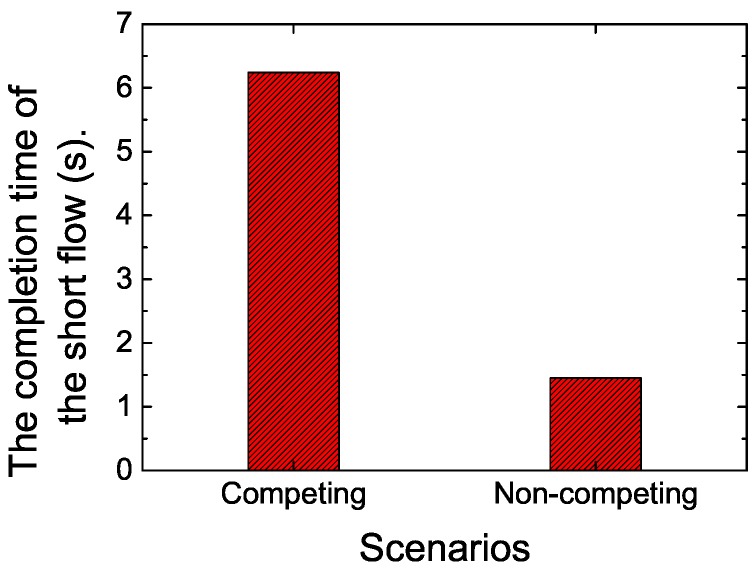
The flow completion time (FCT) of the short flow when it competes with a long flow with the size of 10 MB or not. The size of the short flow is 128 KB.

To validate whether the large buffer size in cellular networks would impact the performance of short flows or not, we calculated the FCT of the short flow in the network scenarios where there were competing long flows or where there were not. The results are shown in Figure 2. In the competing network scenario, a short flow competes with a long flow. The long flow starts at 0 s, and the short flow starts at 30 s.

**Figure 3:**
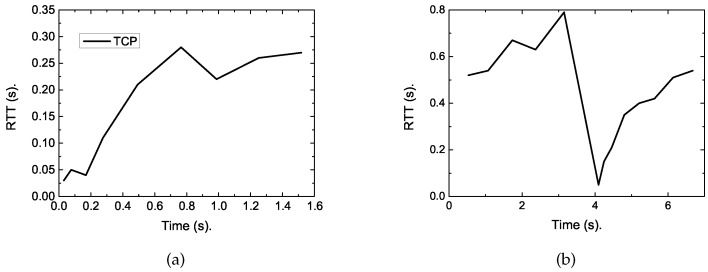
The traced round trip time (RTT) varying with time of the short flow. (**a**) No competing long flow exists, (**b**) The competing long flow exists.

According to the results, the completion time of the short flow increased sharply when the long flow joins. To reveal the reasons, we traced the RTT varying with time and conducted statistics on the packet drop rate. According to Figure 3, the RTT can be up to 800 ms when the long flow exists. The reason lies in that the buffers in cellular networks are oversized, which can absorb a large amount of packets, causing large queuing delay, thus long RTT. This leads to a large FCT of short flows.

What is worse is that the default congestion control algorithm is loss-based, which will not slow down its sending rate until the buffer is full. When the buffer cannot accommodate all the in-flight packets, packet loss occurs. According to our statistics, when there is only one short flow with size of 128 KB, there is no packet lost event. However, when a 10 MB long flow competes with this short flow, the drop rate is up to 2.75%. This will further increase the short flow’s completion time. On the other hand, these lost packets have to be retransmitted, causing wasting of network resource. As validated in Section 5, the obtained network goodput of regular TCP is smaller than the improved algorithms.

Based on the above analysis, we concluded that the oversized buffer in the cellular networks may lead to extremely long delays, causing performance degradation for both short flows and long flows, especially when they are competing for the same wireless access network. The observation motivated us to design a novel approach to effectively control the delay.

## 4. The Proposed DFCSD Algorithm

The goal of the proposed DFCSD algorithm was to control the delay caused by the persistent queueing to improve the TCP performance for both short flows and long flows, especially when both long flows and short flows compete for the same mobile access network. In this section, we first describe the TCP model where users receive data packets through wireless access links, such as 5G and LTE networks. Then, we present DFCSD.

### 4.1. Problem Formulation

We considered a network shared by a set S={1,…,s} of flows. The path of each flow s∈S consists of a set of links l∈L. Every flow *s* maintains its own cwnd $w_{s}$ and transmission rate $x_{s}$. Each flow *s* is associated with utility function $U_{s}\left(x_{s}\right)$ that is assumed to be concave and differentiable. Each link has capacity $c_{l}$. We denote the set of flows that pass through link *l* by $S_{l}$. Let $y_{l}:=\sum_{l\in L}x_{s}$ be the total packet arrival rate at link *l*.

The objective of TCP and its variants is to determine appropriate rates for the flows in order to maximize the total utility subject to link capacity constraints. Thus, we have:

(1) $$\begin{array}{c} Max\sum_{s\in S}U_{s}\left(x_{s}\right)\\ \mathrm{subject}\;\mathrm{to}\;y_{l}\leq c_{l},\;\mathrm{for}\;\mathrm{all}\;\mathrm{links}\;l. \end{array}$$

There exists a unique optimal solution for $x_{s}$, since the objective function is strictly concave and the feasible region is compact [6].

Consider the standard dual problem of Equation (1) and obtain the Lagrangian function as:

(2) $$\begin{array}{c} L\left(x,p\right)\;:\;=\;\sum_{s}U_{s}\left(x_{s}\right)-\sum_{l}p_{l}\left(y_{l}-c_{l}\right)\\ \;=\;\sum_{s\in S}(U_{s}\left(x_{s}\right)-x_{s}p_{s})+\sum_{l}p_{l}c_{l}, \end{array}$$

where the multiplier *p* can be interpreted as the price or the congestion signal, such as queue length and loss probability associated with link *l*, and:

(3) $$p_{s}=\sum_{l\in s}p_{l},$$

is the aggregate price of the links constituting the path of flow *s*. Thus, we call $p_{s}$ the path price.

From the Karush–Kuhn–Tucker conditions (KKT) [37], the optimal $x_{s}$ is achieved when ∇L(x,p)=0, i.e.,

(4) $$U_{s}^{\prime }\left(x_{s}\right)=p_{s}\left(t\right)\;\mathrm{for}\;\mathrm{all}\;s,$$

where Us′(xs)=dU(x)dU(x)dxdx.

The proposed DFCSD algorithm is a receive-oriented approach, and one of its core ideas is to control the number of packets backlogged at the routers, thus reducing the queueing delay, which is similar with TCP Vegas [15]. In TCP Vegas, a source calculates the difference diff between its expected rate $w_{s}\left(t\right)$/$d_{s}$ and its actual rate $w_{s}\left(t\right)$/$D_{s}\left(t\right)$, as shown in Equation (5) where $w_{s}\left(t\right)$ is the cwnd, $D_{s}\left(t\right)$ is the average RTT in the last round, and $d_{s}$ is the minimal RTT that has been measured so far.

(5) $$diff=\frac{w_{s}\left(t\right)}{d_{s}}-\frac{w_{s}\left(t\right)}{D_{s}\left(t\right)}.$$

If $diff<\alpha_{s}$, the cwnd is increased by one packet. If $diff>\alpha_{s}$, the cwnd is decreased by one packet. If the difference is equal to $\alpha_{s}$, the window size is unchanged. Its utility function is [38,39]:

(6) $$U_{s}\left(x_{s}\right)=a_{s}d_{s}logx_{s}.$$

When the algorithm converges the equilibrium windows $w^{*}=\left(w_{s}^{*},s\in S\right)$ and the associated equilibrium RTTs $D^{*}=\left(D_{s}^{*},s\in S\right)$ satisfy:

(7) $$\frac{w_{s}^{*}}{d_{s}}-\frac{w_{s}^{*}}{D_{s}^{*}}=\alpha_{s}.$$

From Equation (7), by multiplying $d_{s}$ and replacing $\frac{w_{s}^{*}}{D_{s}^{*}}$ with $x_{s}^{*}$, we can obtain that:

(8) $$w_{s}^{*}-x_{s}^{*}d_{s}=\alpha_{s}d_{s},$$

which means that the window size $w_{s}^{*}$ minus the BDP $x_{s}^{*}d_{s}$ equals $\alpha_{s}d_{s}$, the total backlog buffered in the path of *s*. In other words, we see that a source increments or decrements its window according to whether the total backlog $w_{s}\left(t\right)-x_{s}\left(t\right)d_{s}$ is smaller or larger than $\alpha_{s}d_{s}$.

In addition, according to Equations (4) and (6), we can also obtain that:

(9) $$U_{s}^{\prime }\left(x_{s}\right)=\frac{\alpha_{s}d_{s}}{x_{s}^{*}}=p_{s}.$$

By substituting $p_{s}=rtt-baseRTT$ into Equation (9), we have:

(10) $$x_{s}^{*}=\frac{\alpha_{s}d_{s}}{p_{s}},$$

which denotes the transmission rate that maximizes the network utility.

### 4.2. The Calculation of the Receive Window

We now focus on typical wireless access network scenarios, where users are connected through 5G and LTE networks. In this case, wireless links often become a bottleneck due to their limited bandwidth. Hence, in wireless access networks, we approximate the sum price as the price of an access link, i.e.,

(11) $$p_{s}\approx p_{last\_hop}.$$

Thus, we can assume that all the flows belonging to the same user in mobile networks have the same price as the base stations typically have a separate buffer space for each user [4,5].

DFCSD is similar to Vegas in the way it adjusts the sending rate to control the number of packets stayed in the buffer, which applies the RTT based flow control at the receiver side. More specifically, the rwnd is incremented or decremented by one packet in the next period by comparing the current rate $x_{s}\left(t\right)$ with the target rate $\alpha_{s}d_{s}/p_{s}$, as shown in Equation (10), and achieves equilibrium when the rate is $\alpha_{s}d_{s}/p_{s}$.

Moreover, it aims to improve the performance of short flows in mobile networks where the buffer is oversized and long delay exists, especially when short flows coexist with long flows. To this end, we should also give short flows higher priority when competing with long flows. Thus, DFCSD provides service differentiation for these flows by setting different $\alpha_{s}$ based on the data amount that has been transmitted. According to Equation (8), a smaller $\alpha_{s}$ means that a smaller number of data amount of flow *s* can be buffered. DFCSD aims to improve the FCT of short flows when they are competing with long flows as they pile up packets at BSs, APs, and end hosts with oversized buffers. Thus, larger flows should have the smaller $\alpha_{s}$ so that short flows can still inject data into the buffer when large flows start to slow down its sending rate.

In addition, the backlog buffered in the path of flow *s* should be no larger than the default maximum value, i.e., $\alpha_{s}d_{s}$. Thus, the $\alpha_{s}$ is in the range $\left[0,\alpha_{s}\right]$ and should have a significant correlation with the flow size, which ranges from 0 to infinity. Based on the above considerations, we define the exponential function as shown in Equation (12) to achieve these goals.

(12) $$\alpha_{s}^{\prime }=\alpha_{s}e^{\left(size_{\min}-size_{s}\right)/\left(size_{\max}-size_{\min}\right)},$$

where $size_{s}$ is the transmitted data amount of flow *s*, and $size_{\min}$ and $size_{\max}$ are the minimum and maximum value of the competing flow sizes.

**Figure 4:**
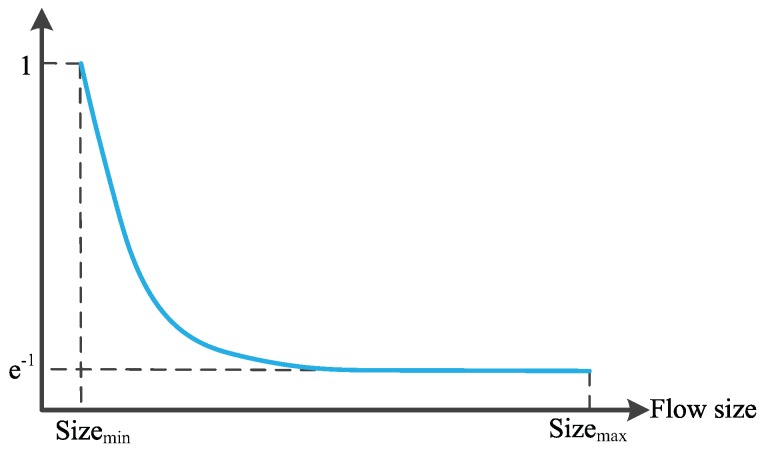
The range of the exponential function used in Equation (12) for the calculation of receive window (rwnd).

According to Figure 4, which draws the curve of $\alpha_{s}^{\prime }$, $\alpha_{s}^{\prime }$ is in the range of [$\alpha_{s}/e$, $\alpha_{s}$] and decreases with the increase of competing flow size. As $\alpha_{s}^{\prime }$ denotes the number of packets buffered in the path of flow *s*, we can get that the smaller the flow is, the more packets that it can be backlogged in the shared buffer. This gives the short flows more opportunities for transmission as long flows start to decrease its data rate when it detects the number of the packets buffered reaches its $\alpha_{s}^{\prime }$. However, the value of $\alpha_{s}^{\prime }$ belonging to the short flow is larger than that of the long flow, as analyzed above. The short flow will continue to increase its sending rate until the the backlogged packets reaches its $\alpha_{s}^{\prime }$. On the other hand, we know that the buffer is mainly occupied by long flows, which carry a large amount of data. Limiting the backlogged packets of the long flow can effectively avoid the high RTT and packet loss caused by buffer overflow, thus increasing the transmission efficiency.

It is important to note that for the flow *s* with the largest size when multiple flows are competing on the network, its $\alpha_{s}^{\prime }$ is minimum and the value is $\alpha_{s}/e$ rather than 0. This setting guarantees that the flow with the largest size can still inject a small number of data in the buffer, with the aim of maintaining the throughput of long flows while controlling the queuing delay.

Above all, according to Equations (10) and (12), the receiver with DFCSD advertises rwnd as:

(13) $$rwnd_{s}=\alpha_{s}^{\prime }\times \frac{D_{s}d_{s}}{D_{s}-d_{s}}.$$

### 4.3. The DFCSD Algorithm

Algorithm 1 shows the details of the DFCSD. According to Equation (13), the receiver should obtain the current RTT, namely, $D_{s}$, to calculate the rwnd. If the TCP timestamp option [5] is available, DFCSD can use it to obtain a more accurate estimation of the RTT (Line 10–12). Fortunately, both Windows Server and Linux support the TCP timestamp option, as long as the client requests it in the initial SYN segment [4,5]. If the timestamp option is available, DFCSD uses the same technique as DRS [40] and DRWA [4,5] to measure RTT on the receiver side (Line 8).

After knowing the RTT, DFCSD records the $d_{s}$, which is the minimum RTT ever seen in this connection and counts the amount of data, $size_{s}$, that flow s has been received. Then, DFCSD sets the rwnd according to Equation (13).

The ideas stem from delay-based congestion control algorithms but work better than they do for two reasons. First, in cellular networks, a base station typically has a separate buffer space for each user [4,5], and it is always the bottleneck. In this condition, DFCSD will not be affected by the flows belonging to other users. Furthermore, DFCSD only guides the TCP cwnd by advertising an adaptive rwnd, and the bandwidth probing responsibility still lies with the TCP congestion control algorithm at the sender. Therefore, typical throughput degradation seen in delay-based TCP will not appear.

**Algorithm 1:** The DFCSD Algorithm

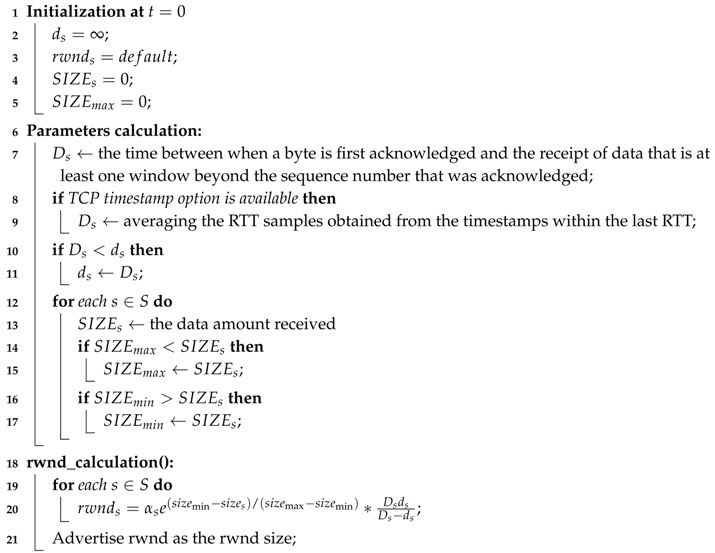



## 5. Evaluation

In this section, we validate our proposed DFCSD algorithm by comparing its performance to regular TCP, the DRWA algorithm [4,5], and the RTAC [6] scheme. The version of TCP is TCP Reno, which is the default value of ns2 and is a loss-based congestion control algorithm.

**Figure 5:**
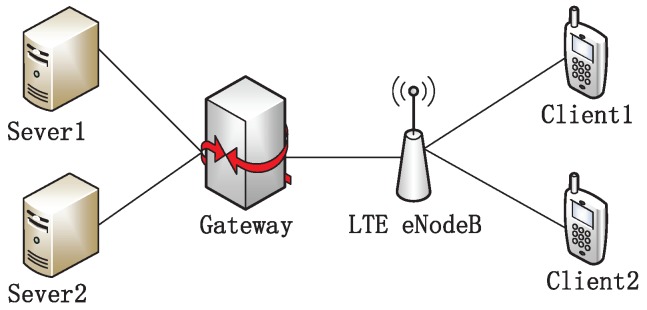
Simulation topology.

Our experiments were conducted on the ns-2 (version 2.35). We used the TCP algorithm embedded in this simulation testbed and implemented the proposed DFCSD and DRWA, as well as RTAC. The topology is shown in Figure 5. Two client devices are associated with the same LTE eNodeB, and download data from the server which is located in the wired side. The eNodeB is attached to a gateway node through the Ethernet with 125 Mbps bandwidth and a 2 ms delay, ensuring it is not the bottleneck of the testbed. The buffer size at the eNodeB is set as the default value of 50 packets in the LTE. The default value of the wireless transmission rate in ns2 is 1 Mpbs. In the last part of the experiment, we conducted the performance of each algorithm with varying wirless transmission rates which vary from 1 Mbps to 54 Mbps.

**Figure 6:**
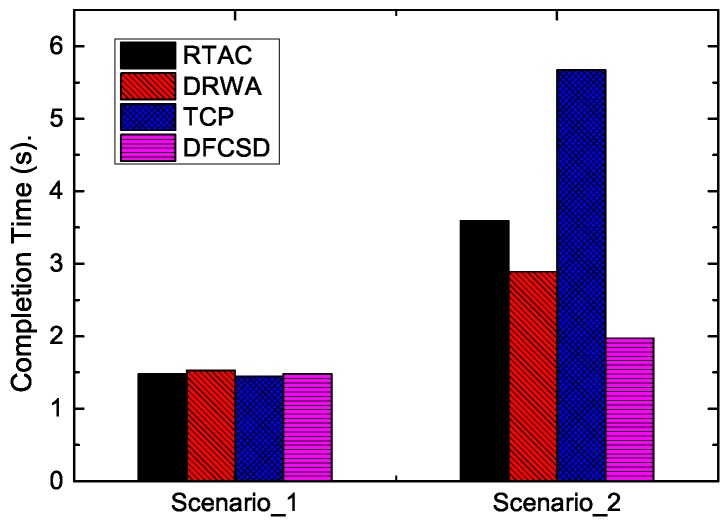
The FCT of the short flow when it competes with a long flow with the size of 10 MB or not. The size of the short flow is 128 KB.

To illustrate the performance impairments for short flows caused by the large buffer size in LTE networks, we first evaluated the completion time of a short flow when there was a concurrent long flow (Scenario_2) or not (Scenario_1) over a mobile device. The size of the long flow and short flow is 10 MB and 128 KB, respectively. The long flow starts at 0 s, and the short flow starts at 30 s. The results are shown in Figure 6.

**Figure 7:**
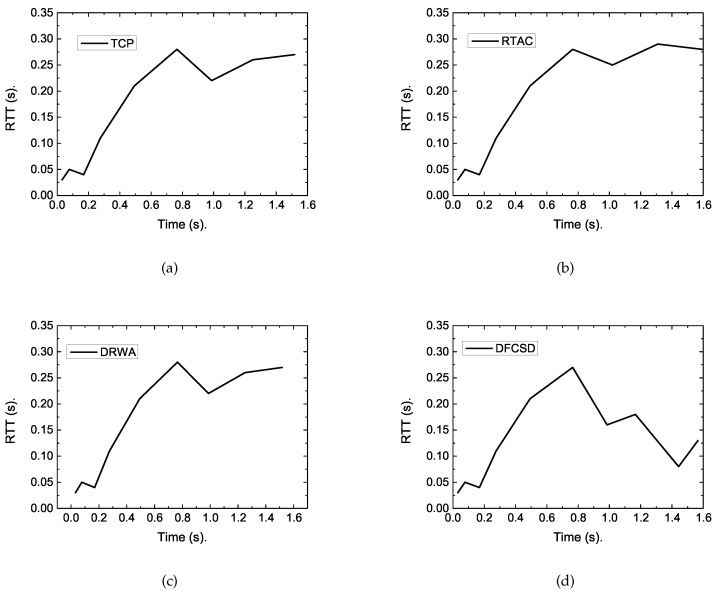
The traced RTT varying with time of each algorithm when only one 128 kB short flow transferred in the network. (**a**) Transmission control protocol (TCP), (**b**) Receiver-side TCP Adaptive queue Control (RTAC), (**c**) Dynamic Receiver Window Adjustment (DRWA), (**d**) Delay-based Flow Control algorithm with Service Differentiation (DFCSD).

According to Figure 6, when there was only one short flow (Scenario_1), the completion time of the four algorithms were nearly the same, with the value of about 1.48 ms, and the RTT of each algorithm differentiated a little with the maximum value of about 300 ms, as shown in Figure 7. However, when the long flow competed with the short flow, the completion time of the short flow increased sharply with default TCP. All the improved algorithms, namely, DRWA, RTAC, and the proposed DFCSD can alleviate this phenomenon, where DFCSD performed best, followed by RTAC.

**Figure 8:**
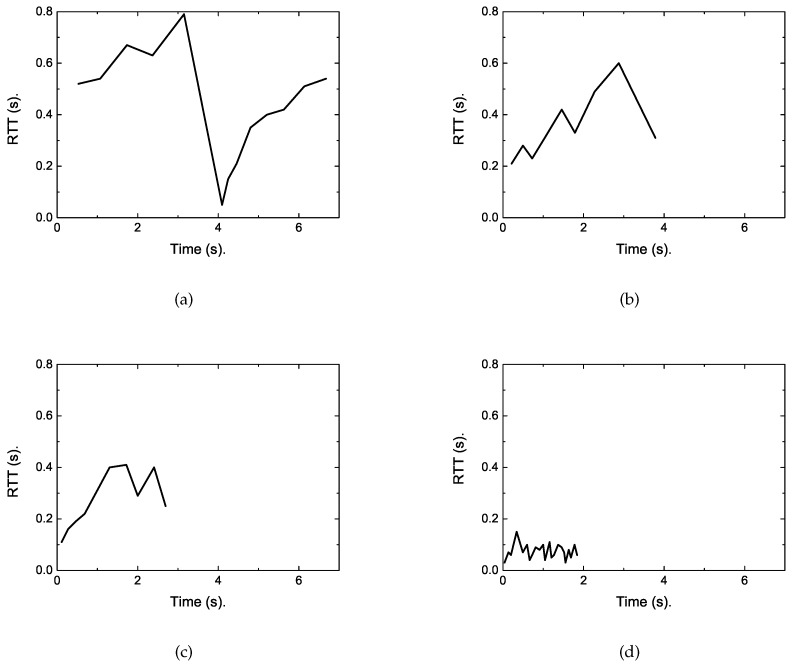
The traced RTT varying with time of each algorithm when a 128 kB short flow competes with a long flow with the size of 10 Mb. (**a**) TCP, (**b**) RTAC, (**c**) DRWA, (**d**) DFCSD.

To reveal the reasons, we traced the RTT of the short flow under each algorithm. The results are shown in Figure 8. As presented in these figures, we can obtain the reason lies in that regular TCP is loss-based, whose cwnd will continue to grow until the buffer size is full, thus packet loss occurs. This can fully utilize the network resource in common network scenarios. However, the buffers of eNodeB in LTE are heavily provisioned to accommodate the dynamic cellular link. This will result in up to 800 ms of round trip delay [4,5], as shown in Figure 8, which is far larger than that depicted in Figure 7 when no competing long flow exists. This causes a large completion time for short flows. However, for the proposed DFCSD algorithm, the sending rate is slowed down when the measured RTT exceeds a certain threshold value, which can control the backlogged packets in the buffer, and thus the RTT.

**Figure 9:**
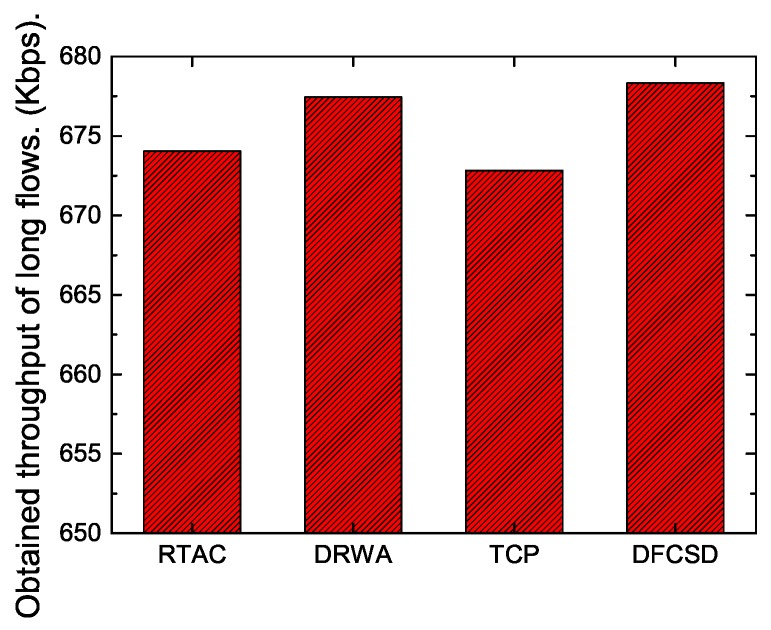
The obtained throughput of the long flows when a short flow competes with a long flow whose sizes are 128 KB and 10 MB, respectively.

**Figure 10:**
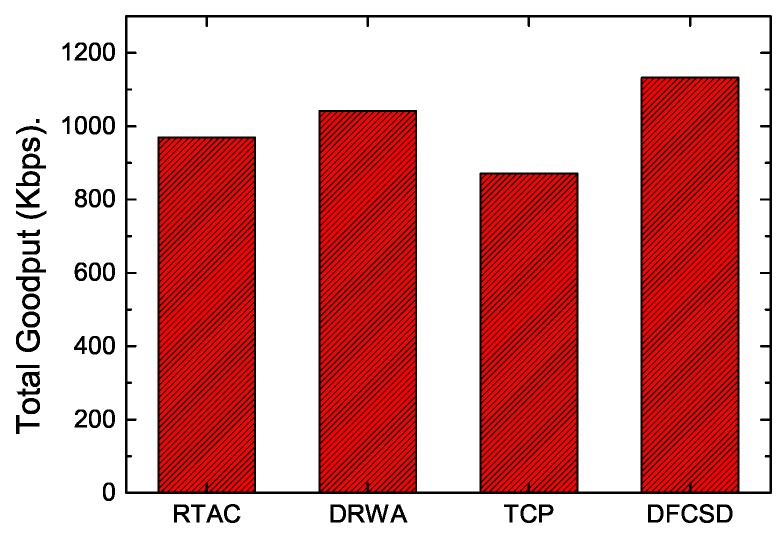
The obtained goodput of the network when a short flow competes with a long flow whose sizes are 128 KB and 10 MB, respectively.

In addition, we also calculated the throughput of long flows and the obtained goodput of the networks to investigate how the algorithms influence them. The results are depicted in Figure 9 and Figure 10. According to Figure 9, there is very little difference (about 5 Kbps) between the throughput of long flows with each algorithm, which validates that the performance improvement of DFCSD for short flows depends on the utilization of the untapped resource rather than suppressing long flows, as the main idea of DFCSD is to alleviate the large queueing delay caused by the oversized buffer size to improve the FCT of the short flows and to avoid buffer overflow which brings retransmissions and even RTOs. The loss-based algorithms, trying to find the limit for a given connection by forcing a router buffer somewhere to overflow, cause packets to be dropped occasionally. The retransmissions caused by the packet loss of each algorithm are shown in Table 1. For DFCSD, the phenomenon does not exist as it slows down its sending rate before the buffer is full, achieving higher network utilization. As a result, the obtained network goodput, shown in Figure 10, reveals that DFCSD outperformed other algorithms, followed by DRWA.

**Figure 11:**
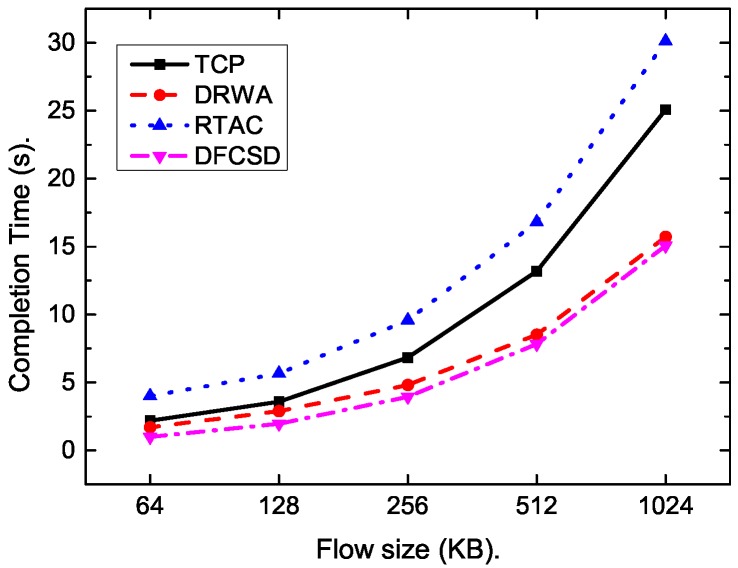
The FCT of the short flow when it competes with a long flow with the size of 10 Mb. The size of the short flow varies from 64 KB to 1024 KB.

**Figure 12:**
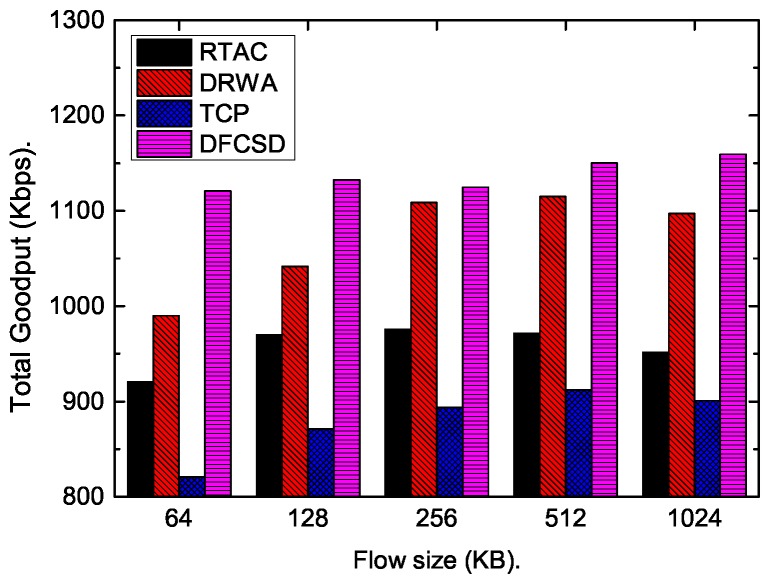
The obtained goodput of the network when the size of the competing short flow varies from 128 KB to 1024 KB.

**Figure 13:**
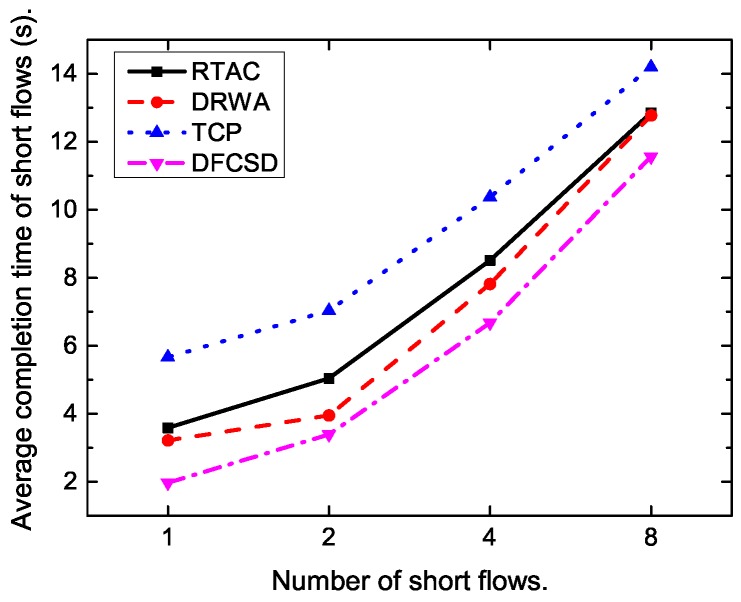
The FCT of the short flows when the number of the concurrent short flows varies from 2 to 8.

**Figure 14:**
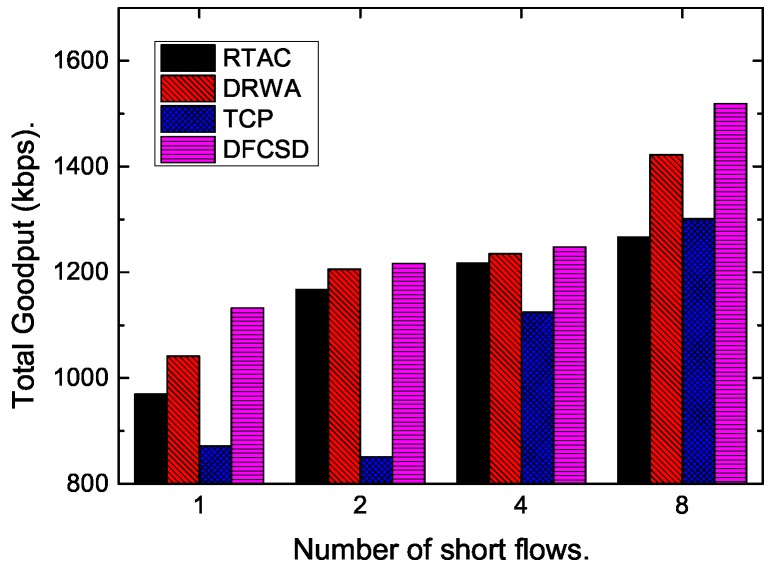
The obtained goodput of the network when the number of the competing short flow varies from 2 to 8.

Then, we further conducted experiments by varying the size, as well as the number, of the short flows. Figure 11, Figure 12, Figure 13 and Figure 14 show the results. The results are consistent with those when only one short flow competing with long flows. More specifically, the average FCT increased with the size of short flows, as well as the number of concurrent short flows. The proposed DFCSD performed best, followed by DRWA, and TCP performed worst. The gains benefit from the controlled queuing delay as analyzed above. On the other hand, for the obtained goodput of each algorithm, TCP also performed worse compared to other algorithms, due to the buffer overflow and the retransmitted packets, wasting the network resource.

**Figure 15:**
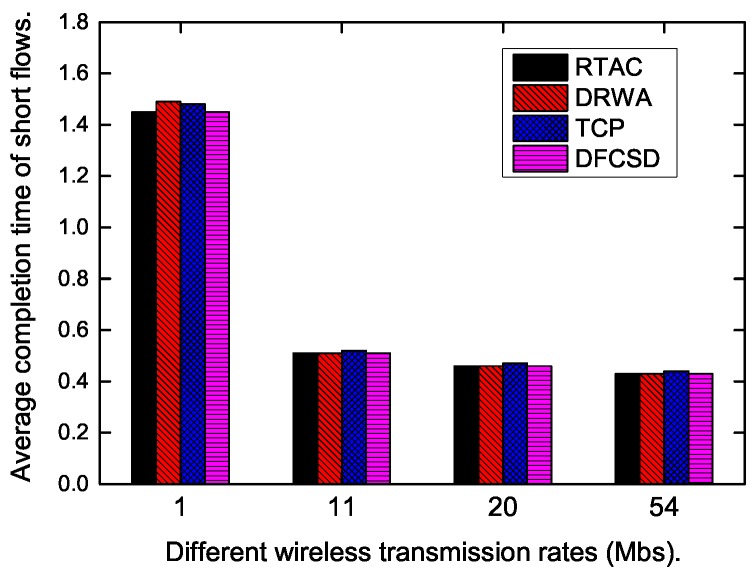
The FCT of the short flows when the wireless bandwidth varies from 1 Mbps to 54 Mbps when there are no long flows.

**Figure 16:**
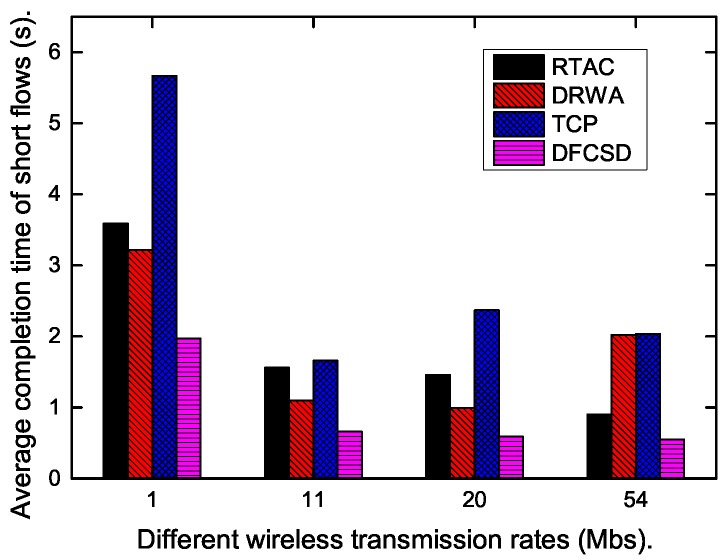
The FCT of the short flows when the wireless bandwidth varies from 1 Mbps to 54 Mbps when long flows exist.

**Figure 17:**
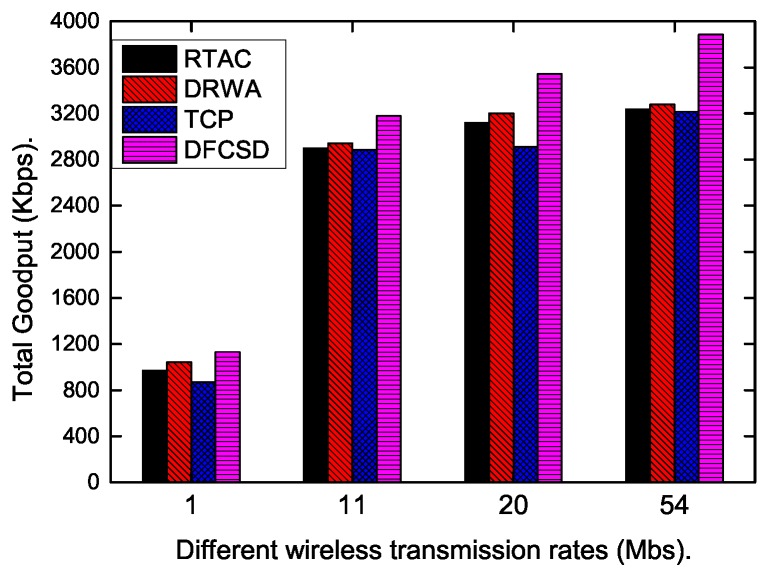
The obtained goodput of the network when the wireless bandwidth varies from 1 Mbps to 54 Mbps.

Finally, we did experiments when the wireless rate varied from 1 Mbps to 54 Mpbs to investigate the impact of the wireless rate on each algorithm. The results are shown in Figure 15, Figure 16 and Figure 17. According to these figures, the completion time of the short flow decreases with increasing wireless rate and increases sharply when long flow exists. The results are consistent with our conclusion that long flows have a significant impact on the performance of the competing short flows. In addition, we also calculated the obtained goodput of the networks in this varying wireless transmit scenarios, which is depicted in Figure 17. DFCSD also outperformed other algorithms, followed by DRWA. As analyzed above, this is a benefit of the reduced retransmissions. The retranmissions are caused by the packet loss and packet retransmission may lead to the waste of the network resource, thus lower network goodput.

## 6. Conclusions and Future Work

The oversized buffer at intermediate routers and the receiver side in cellular networks may lead to long delays, which will significantly influence the performance of short flows. For this paper, we conducted an in-depth study of TCP to find the root reasons and proposed DFCSD to alleviate this issue, which is a receiver side countermeasure. DFCSD first constantly monitors each flow’s status information, and then it adaptively calculates the advertised rwnd of each flow based on the TCP analytical model to control the transmission rate. By limiting the queuing delay for the competing traffic belonging to each device, DFCSD improved the short FCT while maintaining high performance for long-lived flows. The results obtained from extensive experiments demonstrate the effectiveness of DFCSD. Future investigations will focus on exploring an improved TCP algorithm for HTTP, like short flows combining the scheduling algorithms in LTE. In addition, the mobile devices are always equipped with multiple interfaces and multipath TCP has been proposed to fully utilize all the available network resources. How to improve the performance of MPTCP in these resource-competitive environments is also interesting and challenging.

## Figures and Tables

**Table 1:** The amount of data retransmitted of the network in Scenario_2 shown in Figure 6.

Algorithms	Num_Packets_Retransmitted	Drop Rate (%)
RTAC	28	0.274
DRWA	0	0
TCP	92	0.898
DFCSD	0	0

## References

[1] Wang Z., Tan Y., Zhang X. Experimental Evaluation of Modern TCP Variants in MEC-enabled Cellular Networks. Proceedings of the 2018 10th International Conference on Wireless Communications and Signal Processing (WCSP).

[2] Bhuiyan M.Z.A., Wang G., Vasilakos A.V. (2015). Local Area Prediction-Based Mobile Target Tracking in Wireless Sensor Networks. IEEE Trans. Comput..

[3] Gao K., Han F., Dong P., Xiong N., Du R. (2019). Connected Vehicle as a Mobile Sensor for Real Time Queue Length at Signalized Intersections. Sensors.

[4] Jiang H., Wang Y., Lee K., Rhee I. Tackling bufferbloat in 3G/4G networks. Proceedings of the 2012 Internet Measurement Conference.

[5] Jiang H., Wang Y., Lee K., Rhee I. (2016). DRWA: A Receiver-Centric Solution to Bufferbloat in Cellular Networks. IEEE Trans. Mob. Comput..

[6] Im H., Joo C., Lee T., Bahk S. (2016). Receiver-side TCP countermeasure to bufferbloat in wireless access networks. IEEE Trans. Mob. Comput..

[7] Abbasloo S., Tong L., Yang X., Chao H.J. Cellular Controlled Delay TCP (C2TCP). Proceedings of the Ifip Networking Conference.

[8] Henderson T., Floyd S., Gurtov A., Nishida Y. The NewReno Modification to TCP’s Fast Recovery Algorithm. http://www.rfc-editor.org/info/rfc6582.

[9] Ha S., Rhee I., Xu L. (2008). CUBIC: A new TCP-friendly high-speed TCP variant. Acm Sigops Operat. Syst. Rev..

[10] Winstein K., Sivaraman A., Balakrishnan H. Stochastic forecasts achieve high throughput and low delay over cellular networks. Proceedings of the Usenix Conference on Networked Systems Design & Implementation.

[11] Zaki Y., Pötsch T., Chen J., Subramanian L., Görg C. (2015). Adaptive Congestion Control for Unpredictable Cellular Networks. Acm Sigcomm Comput. Commun. Rev..

[12] Huang J., Feng Q., Guo Y., Zhou Y., Qiang X., Mao Z.M., Sen S., Spatscheck O. (2013). An In-depth Study of LTE: Effect of Network Protocol and Application Behavior on Performance. Acm Sigcomm Comput. Commun. Rev..

[13] Robert R., Atxutegi E., Arvidsson A., Liberal F., Grinnemo K.J. Behaviour of Common TCP Variants over LTE. Proceedings of the Global Communications Conference.

[14] Alfredsson S., Giudice G.D., Garcia J., Brunstrom A., Cicco L.D., Mascolo S. Impact of TCP congestion control on bufferbloat in cellular networks. Proceedings of the World of Wireless, Mobile & Multimedia Networks.

[15] O’Malley S.W., Brakmo L.S., Peterson L.L. (1994). TCP Vegas: New Techniques for Congestion Detection and Avoidance. Sigcomm.

[16] Nichols K., Jacobson V., McGregor A., Iyengar J. Controlled delay active queue management. https://tools.ietf.org/html/draft-ietf-aqm-codel-10.

[17] Pan R., Natarajan P., Piglione C., Prabhu M.S., Subramanian V., Baker F., VerSteeg B. PIE: A lightweight control scheme to address the bufferbloat problem. Proceedings of the IEEE 14th International Conference on High Performance Switching and Routing, HPSR.

[18] Hoeiland-Joergensen T., McKenney P., Taht D., Gettys J., Dumazet E. RFC8290: The Flow Queue CoDel Packet Scheduler andActive Queue Management Algorithm. http://www.rfc-editor.org/rfc/pdfrfc/rfc8290.txt.pdf.

[19] Barik R., Welzl M., Ferlin S., Alay O. LISA: A linked slow-start algorithm for MPTCP. Proceedings of the 2016 IEEE International Conference on Communications (ICC).

[20] Atxutegi E., Arvidsson A., Liberal F., Grinnemo K.J., Brunstrom A. (2018). TCP Performance over Current Cellular Access: A Comprehensive Analysis.

[21] Jacobson V. (1988). Congestion avoidance and control. ACM SIGCOMM Comput. Commun. Rev..

[22] Floyd S. (2003). HighSpeed TCP for large congestion windows. IETF RFC 3649.

[23] Wei D.X., Jin C., Low S.H., Hegde S. (2006). FAST TCP: Motivation, architecture, algorithms, performance. IEEE/ACM Trans. Netw..

[24] Dong P., Yang W., Tang W., Huang J., Wang H., Pan Y., Wang J. (2018). Reducing transport latency for short flows with multipath TCP. J. Netw. Comput. Appl..

[25] Wang J., Dong P., Chen J., Huang J., Zhang S., Wang W. (2013). Adaptive explicit congestion control based on bandwidth estimation for high bandwidth-delay product networks. Comput. Commun..

[26] Tan K., Song J. A Compound TCP Approach for High-speed and Long Distance Networks. Proceedings of the 25th IEEE International Conference on Computer Communications (Infocom).

[27] Cardwell N., Cheng Y., Gunn C.S., Yeganeh S.H., Jacobson V. (2017). BBR: Congestion-based congestion control. Commun. ACM.

[28] Rossi D., Testa C., Valenti S., Muscariello L. LEDBAT: The new BitTorrent congestion control protocol. Proceedings of the 19th International Conference on Computer Communications and Networks.

[29] Xu L., Harfoush K., Rhee I. Binary increase congestion control (BIC) for fast long-distance networks. Proceedings of the Ieee Infocom 2004, The 23rd Annual Joint Conference of the IEEE Computer and Communications Societies.

[30] Venkataramani A., Kokku R., Dahlin M. (2002). TCP Nice: A mechanism for background transfers. ACM SIGOPS Oper. Syst. Rev..

[31] Tostes M.V., Fonseca K. TCP TS-Prio: An Approach to end-to-end service differentiation for DiffServ AF classes. Proceedings of the 2006 Advanced International Conference on Telecommunications and International Conference on Internet and Web Applications and Services (AICT/ICIW 2006).

[32] Noureddine W., Tobagi F. Improving the performance of interactive TCP applications using service differentiation. Proceedings of the Twenty-First Annual Joint Conference of the IEEE Computer and Communications Societies.

[33] Floyd S., Jacobson V. (1993). Random early detection gateways for congestion avoidance. IEEE/ACM Trans. Netw..

[34] Feng W.C., Shin K.G., Kandlur D.D., Saha D. (2002). The BLUE active queue management algorithms. IEEE/ACM Trans. Netw..

[35] Kunniyur S., Srikant R. (2001). Analysis and design of an adaptive virtual queue (AVQ) algorithm for active queue management. ACM SIGCOMM Comput. Commun. Rev..

[36] Nichols K., Jacobson V. (2012). Controlling queue delay. Queue.

[37] Boyd S., Vandenberghe L. (2004). Convex Optimization.

[38] Misra V. (2000). Fluid-based analysis of a network of AQM routers supporting TCP flows with an application to RED. Acm Sigcomm Stockh. Swed..

[39] Low S.H. A duality model of TCP and queue management algorithms. https://authors.library.caltech.edu/8574/1/LOWieeeacmtn03.pdf.

[40] Feng W.C., Fisk M., Gardner M., Weigle E. (2002). Dynamic right-sizing: An automated, lightweight, and scalable technique for enhancing grid performance. Protocols for High Speed Networks.

